# Successful Treatment of a Life-Threatening Pulmonary Embolism Following Retroperitoneal Tumor Surgery

**DOI:** 10.7759/cureus.31501

**Published:** 2022-11-14

**Authors:** Noriko Karakida, Shintaro Yanazume, Akio Tokudome, Masahiro Sonoda, Hiroaki Kobayashi

**Affiliations:** 1 Department of Obstetrics and Gynecology, Kagoshima University Hospital, Kagoshima, JPN; 2 Department of Cardiology, National Hospital Organization Kagoshima Medical Center, Kagoshima, JPN

**Keywords:** pelvic tumor, anticoagulant therapy, gynecology, va-ecmo, deep vein thrombosis, retroperitoneal tumor, pulmonary embolism, multidisciplinary treatment, hemorrhage, continuous pressure drain

## Abstract

We encountered a case of life-threatening pulmonary embolism (PE) after an extensive retroperitoneal tumor (RT) surgery. The patient complained of abdominal distension. Preoperatively, an ovarian tumor and colon adenoma were suspected. Upon laparotomy, tumor resection and partial rectal resection were performed; the tumor had originated from the retroperitoneum. On postoperative day 11, the patient suddenly went into fatal shock complicated by strong back pain and dyspnea after the continuous pressure drain was removed. Thrombolysis, anticoagulation, and percutaneous catheter-directed treatment were attempted for the massive PE; however, these induced copious intra-abdominal bleeding. A substantial blood transfusion was required, which increased her body mass by 40 kg. On day 22, an intra-abdominal embolism was resected, and hemodynamics stabilized. RTs have a potential risk of perioperative thromboembolism; therefore, we suggest that surgery should take place in an academic hospital with an experienced circulatory team. To preserve life after PE, early diagnosis and multidisciplinary treatment are indispensable.

## Introduction

Pelvic retroperitoneal tumors (RTs) are relatively rare tumors that are sometimes unexpectedly found by gynecologists. Among pelvic tumors in women, especially ovarian neoplasm is known to be the main cause of deep venous thrombosis and pulmonary embolism (PE), and adequate perioperative thromboprophylaxis is required [1]. Meanwhile, acute severe PEs have a high mortality rate; however, early diagnosis and early treatment are the most important factors for the preservation of life [2]. Nevertheless, unlike ovarian neoplasms, there is still a lack of information regarding the relationship between RTs and thromboembolism.

Here, we report a case of a giant retroperitoneal borderline malignant tumor that caused a massive PE after surgery; the patient survived owing to multidisciplinary treatment. We detail the processes leading to a successful outcome and suggest it is worthwhile to review the etiology of PE in RTs.

## Case presentation

**Figure 1:**
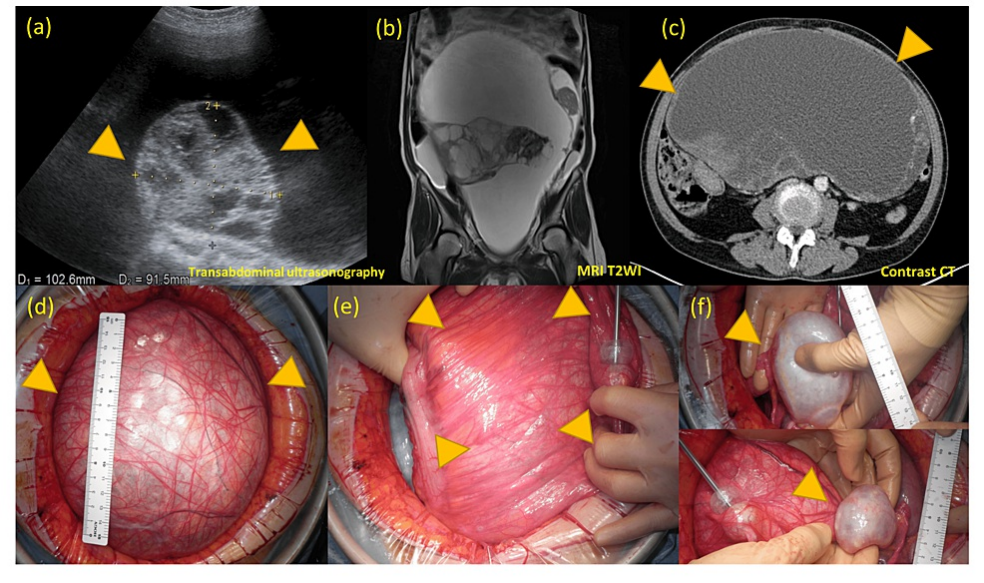
Pre-operative images of the computed tomography and laparotomy findings. (a, b, c) A gigantic multilocular cystic mass with a maximum diameter of 32 cm containing a solid mass. (d, e, f) The huge mass originated from the retroperitoneum; the bilateral ovaries were normal.

Full, informed consent was obtained from the patient. This study was conducted in accordance with the Declaration of Helsinki (as revised in Fortaleza 2013). The patient, a 64-year-old woman, was referred to our hospital with a history of abdominal distension and increased vaginal discharge beginning 18 months previously. A gigantic multicystic tumor was found in the pelvis, and ovarian cancer was suspected. Her body height was 158 cm, and her body weight was 48 kg. Examination of imaging revealed a 32 cm-sized (maximum diameter) giant multilocular cystic tumor involving a solid part in the abdominal cavity (Figure 1). A coagulation profile showed that D-dimer was slightly rising to 1.24 μg/ml. However, no preoperative deep venous thrombus (DVT) was observed on ultrasonography or contrast CT. A detailed evaluation of the pelvic vessels was difficult due to tumor compression. An adenoma (low grade, group 3) was found following a colon endoscopy; therefore, we planned to perform both gynecological and gastroenterological surgery.

We performed a laparotomy and observed that the huge tumor was derived from the left retroperitoneum rather than the ovary; normal bilateral ovaries and uterus were found (Figure 1). Tumor resection and hysterectomy, bilateral salpingo-oophorectomy, omentectomy, external iliac lymph node biopsy, and partial colectomy with end-to-end anastomosis were performed without a colostomy. The tumor’s fluid content was 6700 ml, and the weight was 2985 g. Surgery time was 3 hours and 33 minutes, and blood loss was 235 ml. An intraoperative blood transfusion was not required. The final pathological diagnosis of the tumor was a solitary fibrous tumor of the retroperitoneum with borderline malignancy. The rectum tumor was diagnosed as a tubulovillous adenoma.

To prevent deep vein thrombosis, elastic stockings and a foot pump were used in the perioperative period. With the patient being in a high-risk group for venous thromboembolism, starting two hours after the surgery, low molecular weight heparin (10,000 U/day) was initiated for 24 hours. Enoxaparin sodium (2000 U × 2/day) was injected five days after surgery. The patient was treated according to deep vein thrombosis prophylaxis guidelines in Japan.

The patient recovered uneventfully until an event occurred on day 11 post-surgery. After her abdominal drain was removed, she felt dizzy, and her blood pressure dropped to 75/52 mmHg. Five hours later, she suddenly went into fatal shock, complicated by strong back pain, dyspnea, and peripheral coldness; her blood pressure was also unmeasurable. She was immediately taken to the intensive care unit (ICU), and dopamine hydrochloride and heparin were administered. Shortly after entering the ICU, cardiopulmonary arrest (CPA) occurred, and cardiopulmonary resuscitation (CPR) took place. She was put on an artificial respirator and entered the catheter room for the introduction of veno-arterial extracorporeal membrane oxygenation (VA-ECMO). Cardiac ultrasound showed right ventricular and right atrial dilatation, suggesting severe pulmonary hypertension (the estimated systolic pulmonary artery pressure was 58 mmHg). We suspected PE and performed pulmonary angiography, which revealed a massive thrombus floating in the bi-pulmonary artery.

**Figure 2:**
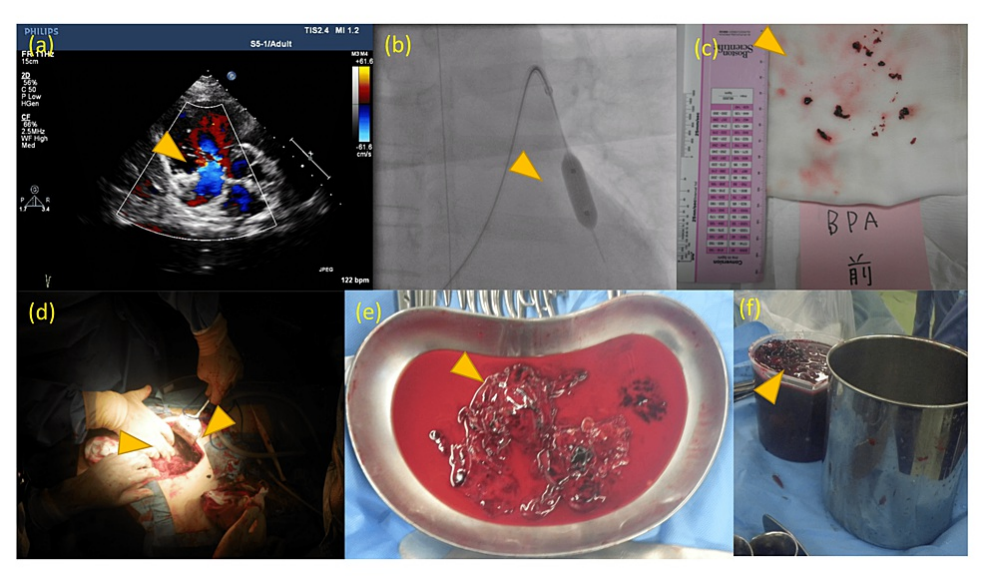
The image of pulmonary embolism treatment. (a) Cardiac ultrasound examination at onset. Right ventricular pressure load was accepted. (b, c) Trans-catheteric removal of the embolism was attempted on day 14. (d, e, f) Intra-abdominal embolism was surgically removed on day 22. Approximately 3920 g of hematoma was removed.

**Figure 3:**
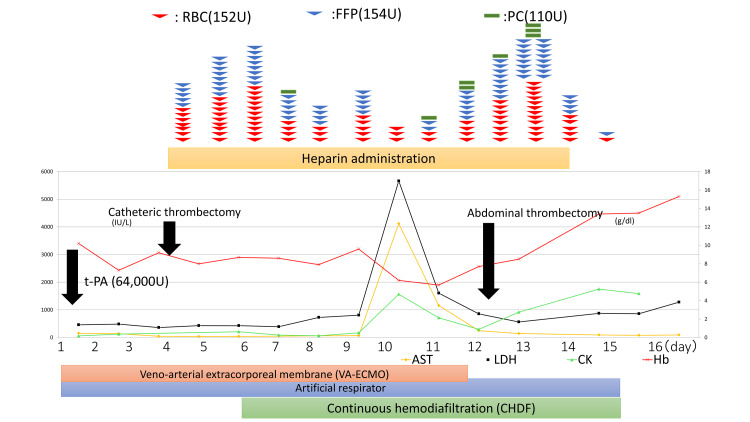
The clinical course of pulmonary embolism treatment. RBC, red cell concentrate; FFP, fresh frozen plasma; PC, platelet concentrate; t-PA, tissue plasminogen activator; AST, aspartate aminotransferase; LDH, lactate dehydrogenase; CK, creatine kinase; Hb, hemoglobin.

We started thrombolysis with t-PA (64 000 U) and anticoagulation with heparin. However, hemodynamic improvement was poor, and thrombectomy was considered. Surgical pulmonary embolectomy was too risky, so percutaneous catheter-directed treatment was performed on day 4, and her hemodynamics improved temporarily. However, the thrombolysis-induced copious intra-abdominal and subcutaneous bleeding persisted. As a result, cardiac output could not be maintained due to intra-abdominal bleeding putting pressure on the vessels and decreasing venous return. To maintain her hemodynamics, she required substantial daily blood transfusions and continuous hemodiafiltration (CHDF) management. The total blood transfusion amounted to RCC (152 U), FFP (154 U), and PLT (110 U). Her weight increased to 88 kg from 48 kg after the blood transfusion; a notable increase of 40 kg. We planned to perform a thrombectomy after discontinuing VA-ECMO with an understanding of the high risk of death. On day 12, intraperitoneal thrombectomy was performed immediately following the discontinuation of VA-ECMO during surgery. In the laparotomy, a large amount (3920 g) of intra-abdominal hematoma was removed (Figure 2). Following this surgery, the patient's circulatory dynamics gradually stabilized, and CHDF and the ventilator were discontinued on day 15 after onset (Figure 3). She underwent rehabilitation for approximately three months and was discharged without any recurrence of thrombosis or RTs.

## Discussion

This case shows that an RT has the potential to cause a fatal perioperative PE. Immediate cardiopulmonary resuscitation and prompt intensive treatment were provided by our academic cardiovascular treatment team. Therefore, we suggest that cases at high risk of PE, including RTs, should be managed in a specialized cardiovascular facility, even if there are no thrombosis-related preoperative abnormalities.

RTs that arise from mesenchymal connective tissues are relatively rare, while malignant RTs are more common [3]. These tumors develop insidiously because of their anatomic location, and 50% of RTs are larger than 20 cm (maximum diameter) at diagnosis [4].

The correlation between RTs and thromboses is not understood. In addition to abdominal symptoms, it has been reported that RTs may be detected by thrombosis [5,6]. PE-related RTs have been mostly reported in the urological field, and a testicular tumor thrombus is the most common cause of PE thrombi rather than a lower limb thrombus [5]. There are no previously reported cases of an RT treated by a gynecology department that recorded the presence of a massive PE. It is difficult to make an accurate preoperative diagnosis of RTs, and the surgery is often undertaken in the process of gynecology [7]. However, previously recorded surgeries have been problematic due to massive hemorrhaging owing to tumor hypervascularity or extensive adhesion with surrounding organs [8].

PE has increased 2.25-fold in the past 10 years in Japan [9]. Acute PE has a 30% mortality rate in untreated cases that have been diagnosed; however, when there is early diagnosis and treatment, the mortality rate is lowered to 2-88% [10]. In the guidelines for PE and DVT (JCS2017) [11], obesity, estrogen treatment, advanced age, malignant diseases, severe infections, etc., are indicated as risk factors for venous thromboembolism. Regarding gynecological patients, a body mass index≧35, abdominal hysterectomy, increased lifetime surgical duration, cancer, age, and transverse tumor diameter have been reported as risk factors [12,13]. Although immediate anticoagulant and thrombolytic therapy for the treatment of acute PE is important, postoperative bleeding remains a risk for patients.

In this case, ovarian cancer was suspected in the preoperative diagnosis, and a laparotomy was performed. The huge tumor was unexpectedly derived from the retroperitoneum, but it could be removed completely without any problem. Age, tumor size, and borderline malignancy were risk factors for DVT and PE. The preoperative examination did not reveal thrombosis, and we took maximum preventive measures by considering the patient to have a high risk of venous thromboembolism. Possible reasons why the patient developed the potentially fatal PE include that her large pelvic RT directly pressed upon the retroperitoneal vessels, causing more complications than intraperitoneal tumors alone. Second, continuous negative pressure drains may have affected the development of severe thrombosis. An abdominal drain was required until the 11th day postoperatively because of the partial colectomy. It has previously been reported that a continuous suction drain after retroperitoneal lymphadenectomy for a gynecological tumor may increase DVT [14].

The diagnosis of PE and multidisciplinary intensive treatment were initiated promptly; however, the postoperative period of the giant retroperitoneal tumor resection caused a large amount of intra-abdominal bleeding. The most difficult management for anticoagulant therapy is coexistence with the risk of bleeding. We, therefore, decided to perform a laparotomy to remove the intra-abdominal thrombi and succeeded in preserving her life.

## Conclusions

Although RTs are relatively rare tumors, gynecologists sometimes encounter them unexpectedly. Compared to an intraperitoneal tumor such as an ovarian tumor, a retroperitoneal tumor is associated with a higher risk of thrombosis; therefore, it is critical for clinicians to pay close attention to perioperative thrombosis. As RTs have the potential to become new risk factors for thromboembolism, it is recommended that they are treated by an experienced team of surgeons, including a cardiologist and an emergency specialist, thus employing a multidisciplinary approach.
